# Construction of a simulation scenario and a low-cost simulator for teaching thoracentesis procedural technique: a validation study

**DOI:** 10.1186/s12909-025-07381-7

**Published:** 2025-07-01

**Authors:** Roberson Antequera Moron, Raphael Raniere de Oliveira Costa, Victor Cardozo, Carlos Ferreira dos Santos, Marcos Antonio Marton Filho, Alessandra Mazzo

**Affiliations:** 1 Bauru School of Dentistry (FOB) and Hospital for Rehabilitation of Craniofacial Anomalies (HRAC), University of São Paulo, R. Silvio Marchione, 3-20 - Vila Nova Cidade Universitaria, Bauru - SP, Sao Paulo, 17012-900 Brazil; 2 Medicine Course, Multicampi Medical Sciences School (EMCM), Federal University of Rio Grande do Norte (UFRN), Caicó, Rio Grande do Norte Brazil; 3 Bauru School of Medicine, University of Sao Paulo, Bauru, Sao Paulo Brazil

**Keywords:** Simulation training, Validation study, Low-cost technology, Thoracic surgical procedures, Cost, Undergraduate

## Abstract

**Background:**

Thoracentesis yields valuable insights in pleural effusion diagnosis by accurately interpreting pleural fluid analysis results and can cause several complications, which emphasizes the importance of training in a simulated environment. There are many expensive simulators related to this procedure and few validated scenarios. This study aimed to build and validate a low-cost simulator and a clinical simulation scenario for teaching the thoracentesis procedural technique to undergraduate medical students.

**Methods:**

This is a methodological study carried out at a public university in the interior of the state of São Paulo, Brazil. It was carried out in three methodological stages, namely: 1) Construction of the simulator, which involved planning, surveying, pricing, and use of material resources 2) Construction of a simulated thoracentesis scenario, based on literature and a simulation script and 3) Validation by experts and pilot study of the simulator and scenario. Experts were selected according to Fehring criteria.

**Results:**

The simulator proved to be suitable and low-cost (US $18). Modifications to the scenario were suggested by the experts and students in the pilot study, with 100.0% agreement.

**Conclusions:**

In conclusion, a low-tech, handmade, and low-cost simulator was built and evaluated for training in the thoracentesis procedural technique, as well as a clinical simulation scenario for the management of patients with pleural effusion, which can be included in various medical teaching contexts.

## Introduction

Throughout the history of surgical education, new challenges, such as a steep learning curve for minimally invasive surgery, and the stronger ethical implications of learning on patients, have made the traditional logic of “*See one, Do one, Teach one*” more problematic, fostering research for novel alternatives [1]. Therefore, ensuring adequate exposure to surgical training during medical graduation became a relevant concern among medical schools [2, 3], and this also applies to the training of procedural techniques [3].

In this context, relevant discussions – such as the European Working Time Directive [4] – have been tensioned to question the traditional quality, quantity, and need for training in procedural technique education. From this perspective, there has been much discussion about the issues that justify the creation and implementation of structured procedural training programs in undergraduate curricula [5].

In procedural training, simulation-based learning is an important step towards a safer and more efficient generation of doctors [6]. Current evidence shows that incorporating the use of animal tissue or other simulation methods into undergraduate educational programs can be a catalyst for advancing education on procedural techniques [7]. Simulators, regardless of whether they use animal tissue (*wet lab*), non-animal devices (*dry lab*), or even corpses, allow for different levels of fidelity that can be adjusted to the expectations of procedural teaching. Lower-fidelity, low-cost modules would be aimed at basic procedural training, while higher-fidelity, high-cost modules can be reserved for teaching more advanced students [7, 8]. When simulators are associated with surgical environment contexts and issues involving decision-making, they can significantly improve the quality of care provided [9].

The use of simulation-based learning involves investments in teacher training, the acquisition of simulators of different technological levels, and the acquisition and/or development of instruments and other technologies. Especially among developing countries, these concerns can contribute to widening inequalities in health training and the tendency to use traditional teaching and learning methods [10].

Among the various skills required by a surgeon and general practitioners, given their frequency in practice and clinical relevance to the patient’s prognosis, is thoracentesis. Thoracentesis is a procedural technique that aims to remove fluid or air from the pleural space, usually with a needle, for diagnosis or relief of respiratory symptoms [11].

Thoracentesis can cause several complications, which emphasizes the importance of training in a simulated environment. The main complications of thoracentesis include pneumothorax, hemothorax due to injury to intercostal vessels, re-expansion edema, pain at the puncture site, empyema, soft tissue infection, and accidental puncture of abdominal viscera such as the liver and spleen. Pneumothorax is the most common complication, occurring in up to 20% of cases, related to medical inexperience, large-gauge needles, excessive extraction of pleural fluid, multiple punctures, chronic obstructive pulmonary disease, repeated thoracenteses, and pleural loculations. Despite this, thoracentesis is considered a less invasive procedure and is the method of choice for obtaining pleural fluid samples, to improve the chance of diagnosis and minimize risks [12].

Clinical simulation can be a viable and reliable strategy for learners to achieve competencies and skills in the subject. In a simulated clinical environment, curricula with simulated thoracentesis practice increased residents’ skills, appreciation of training, evaluation, and feedback [13]. At its most relevant degree of impact on practice [14], evidence shows that prior thoracentesis training of residents improves patient care outcomes [15]. Therefore, recognizing the benefits of clinical simulation for teaching this technique justifies its inclusion in curricula and simulation programs.

The cost of a commercially available thoracentesis simulator ranges from $2,500 to $4,000 [16], not to mention they often require replacement parts [17]. With this in mind, many studies have already presented the validation of low-cost simulators and simulation models for teaching and training the thoracentesis or chest tube insertion by different practitioners, with an average $100 budget — which qualifies as low-cost [16–22]. However, their applicability often relies on advanced technology such as 3D printing [16] or is limited to a context unrelated to undergraduate medical practice, such as nursing [17, 18] or emergency medicine [19–22].

In this context, this study aimed to build and validate a low-cost simulator and a clinical simulation scenario for teaching the thoracentesis procedural technique to undergraduate medical students.

## Material and methods

This is a methodological study carried out at a public university in the interior of the state of São Paulo, Brazil. Methodological research involves three processes, namely technology development, production, and construction Polit and Beck (2011) [23]. The methodological steps of this study are presented in three stages, namely:

### Stage 1—Development and construction of a low-cost simulator

To develop and build the low-cost simulator, the research team asked the following question: What attributes are needed to build a low-cost simulator for teaching the thoracentesis procedural technique?

- Realism: the simulator should provide an experience that can reproduce thoracentesis and pleural drainage procedures as realistically as possible, taking into account the correct techniques for carrying them out, as well as tactile and visual sensory experiences.
- Reproducibility: the simulator should allow for multiple uses and easy replacement of disposable parts once it has reached its useful life.
- Low cost: the simulator should have lower production and maintenance costs than those quoted for prefabricated simulators available for the same purpose.

**Fig. 1:**
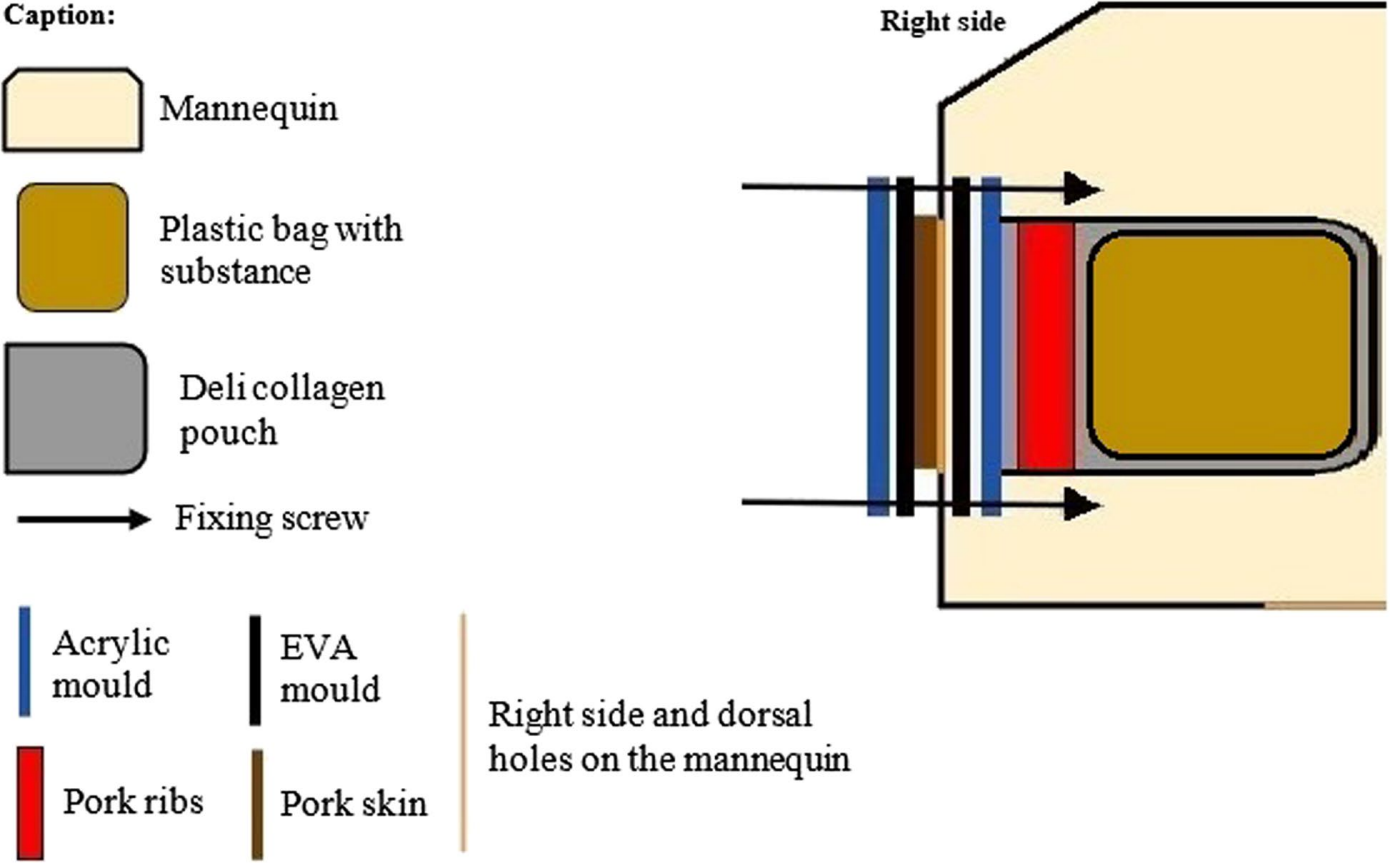
Schematic model of the simulator, Bauru, 2023

After defining the attributes, a search was made for materials in the local trade (Table 1) and a prototype drawing of the simulator’s structure was made (Fig. 1).

**Table 1:** Researched and justified material resources according to the established criteria, Bauru, 2023

**Item description**	**Item photo**	**Quantity/unit price**	**Total US$**
Polythene mannequin in the shape of a male bust.**		05 - US$ 11.8	US$ 59.0
Acrylic plate with 4 screw holes.*		10 - 1.0	10.0
Piece of pork rib measuring 20X12 cm, rectangular in shape, with at least 5 ribs.*		3 - 5.0	15.0
Collagen sausage casing.*		5 - 0.44	2.2
Ethyl Vinyl Acetate (EVA) mould.*		5 - 0.6	3.0
Plastic bag with sealed puncture fluid.*		5 - 0.3	1.5
Boiled pork skin.*		5 - 0.4	2.0
M4X8mmX20mmale and female handle screw**		20 - 0.41	8.2
*Obtained in the local market; **Obtained in https://www.mercadolivre.com.br/

### Stage 2—Building the clinical simulation scenario

To build the clinical simulation scenario, we used the criteria of Fabri et al. (2017) [24], which consider the following structuring elements: the student’s prior knowledge; learning objective; theoretical basis of the activity; preparation of the scenario; and evaluation.

Initially, intending to encourage discussion about its construction, the research team started with the following inquiry: “What information, based on the structuring elements adopted, is essential for the construction of a pleural effusion clinical simulation scenario with an indication for thoracentesis?”.

Two specialists in clinical simulation and two medical-surgical lecturers took part in its development. The scenario included patient care in the environment of an emergency care unit. It took into account the cognitive, affective, and psychomotor domains [25] and was based on evidence [12, 26–32].

### Stage 3—Clinical simulation scenario and simulator validation processes

#### Face and content validation

The criteria proposed by Fehring (1987) [33] were used to select the experts, with a minimum score of five points out of a total of 14: master’s degree (4 points), master’s dissertation in the area of interest of the study (1 point), doctoral thesis in the area of interest of the study (2 points), clinical practice with one year or more of experience in the subject of the study (1 point), specialization in the subject of interest of the study (2 points), publication of relevant research in the area of interest of the study (2 points) and publication of an article in the area of interest of the study in reference journals (2 points). In this study, the area of interest was clinical simulation and the topic of interest was surgical medicine. Three judges took part (100.0%) [33].

Next, to organize the final material, the Delphi Technique was used, which consists of a systematized method for judging information, aimed at obtaining a consensus from experts and users on a given topic, employing validations articulated in phases or cycles. The judges summarize, point out improvements, and consider the material examined in all its dimensions [34].

To assess the consensus between the judges, as well as the degree of agreement between them, the Content Validity Index (CVI) was used, where each item was assessed separately, followed by the instrument as a whole. To do this, two Likert scales were used with a score of one to five, where four and five corresponded to positive responses, three to neutrality, and two and one to negative responses.

The instrument’s index score was calculated using the Content Validity Index (CVI). A minimum value of 80% was set for both the CVI and the agreement index.

#### Pilot study

To adapt the technologies built (simulator and simulated scenario), a pilot test was carried out with undergraduate medical students regularly enrolled in the 4 th semester of the course. The students were invited online by a student researcher. The first ten students enrolled were included in the pilot. The pilot followed the classic cycle of simulated practice which consisted of skills training, scenario development, feedback, debriefing, and evaluation.

Before the skills training and scenario development, the students took part in a dialogue lecture on the subject. They then took part in group skills training, followed by a simulated scenario, debriefing, and feedback. After the simulated practices, the students, in a meeting with the researchers, expressed their opinions on the simulator and on the simulation scenario through an instruments developed by the researchers, and suggested improvements to the practices offered.

The instruments for evaluating the simulator were set in two tables. The first instrument, used to assess the satisfaction of the students was a 6 × 17 table containing 17 items evaluating different qualities of the simulated context: 1. Overall satisfaction with the hands-on practices; 2. The lessons learnt; 3. Motivation when coming to the hands-on practices; 4. The dynamism of the hands-on practices; 5. Active participation in the scenarios developed; 6. Interaction with colleagues; 7. Interaction with lecturers; 8. Satisfaction with the degree of difficulty of the scenarios; 9. Productivity during hands-on practices; 10. Realism of the simulator; 11. Credibility during the scenario; 12. Quality of the material used in the hands-on practices; 13. Quality of the equipment used in the hands-on practices; 14. Quality of the simulators; 15. Satisfaction with the post-scenario discussion (debriefing); 16. Link between scenarios and theory; 17. Appropriateness to the themes developed in the TP classes. The pilot test was carried out in the skills and simulation laboratory at the researchers’ institution.

### Analysis and presentation of results

The results were presented in the form of tables, figures, and a discursive report. After the final analysis of the expert evaluations and the pilot project, the final version of the products was produced.

### Ethical aspects

This study was submitted to the Research Ethics Committee of the Bauru School of Dentistry and was approved under numbers 5.072.385 and 5.294.181. Acceptance to participate in the study was formalized by signing the informed consent form. As this study presents some qualitative aspects of the feedback approach, the Consolidated Criteria for Reporting Qualitative Research (COREQ) checklist was followed [35].

The biological materials used in this study were commercially obtained from supermarkets or meat-packing facilities within the municipality. These materials had already undergone processing by the food industry and, therefore, do not constitute animal research material. The handling and disposal of materials comply with national regulations established by the National Health Surveillance Agency (ANVISA).

## Results

### Building the low-cost simulator

To better analyze the results and have enough training material available for the students, five simulators for thoracentesis training were built identically. The estimated cost of each simulator was 18 US$. A male surgeon took five hours in the making of all the components to be assembled into one simulator. Two technicians (one male and one female) took one hour each into assembling the components in the day of the pilot test.

To enhance the reproducibility of the simulator and to reduce its cost of acquisition and maintenance, plastic mannequins from the fashion retail market were used. The back of the mannequins was cut with a mini-drill saw ® to allow access to the inside. Next, the place where the underarm side wall would be drilled was marked to allow the ribs to be placed. The sealing gasket made from ethylene–vinyl acetate (EVA) was used as a marker at the height of the nipple line. The seal was obtained by interposing EVA and acrylic seals with 4 holes for the screws to pass through. Laser cutting was used to ensure the accuracy of the EVA and acrylic cuts. The walls of the sealing gasket and the fixing acrylic were 2 cm wide and 3 mm thick. The rectangular acrylic joint to allow the ribs to be fitted was made to measure 16.5 X 9 cm.

The pork skins and ribs used were cut using the same acrylic plate as a size mould. The mannequin parts were stored in a freezer (temperature −3° C) and were only assembled on the day of the skills training and development of the simulated scenario, to avoid degradation of the biological components.

To faithfully mimic the sensation of puncturing the pleural space, we used pork ribs bought from a supermarket. The ribs were cut and perforated, while still frozen, to the same size as the mannequin’s “window”, respecting the same direction of inclination as the human ribs. In addition, pork skin of the same size was boiled in water at 100 degrees for 10 min to soften it and improve the feel of human skin. The skin was also perforated at 4 points for fixation.

Internally, the “pleural space” was constructed using a collagen deli bag, with its upper end open, making it possible to change the contents without disassembling the whole simulator each time it was used. The bags were made of plastic, and vacuum-sealed. A volume of 300 mL was added into each bag, enough to be used in five simulated trainings.

**Fig. 2:**
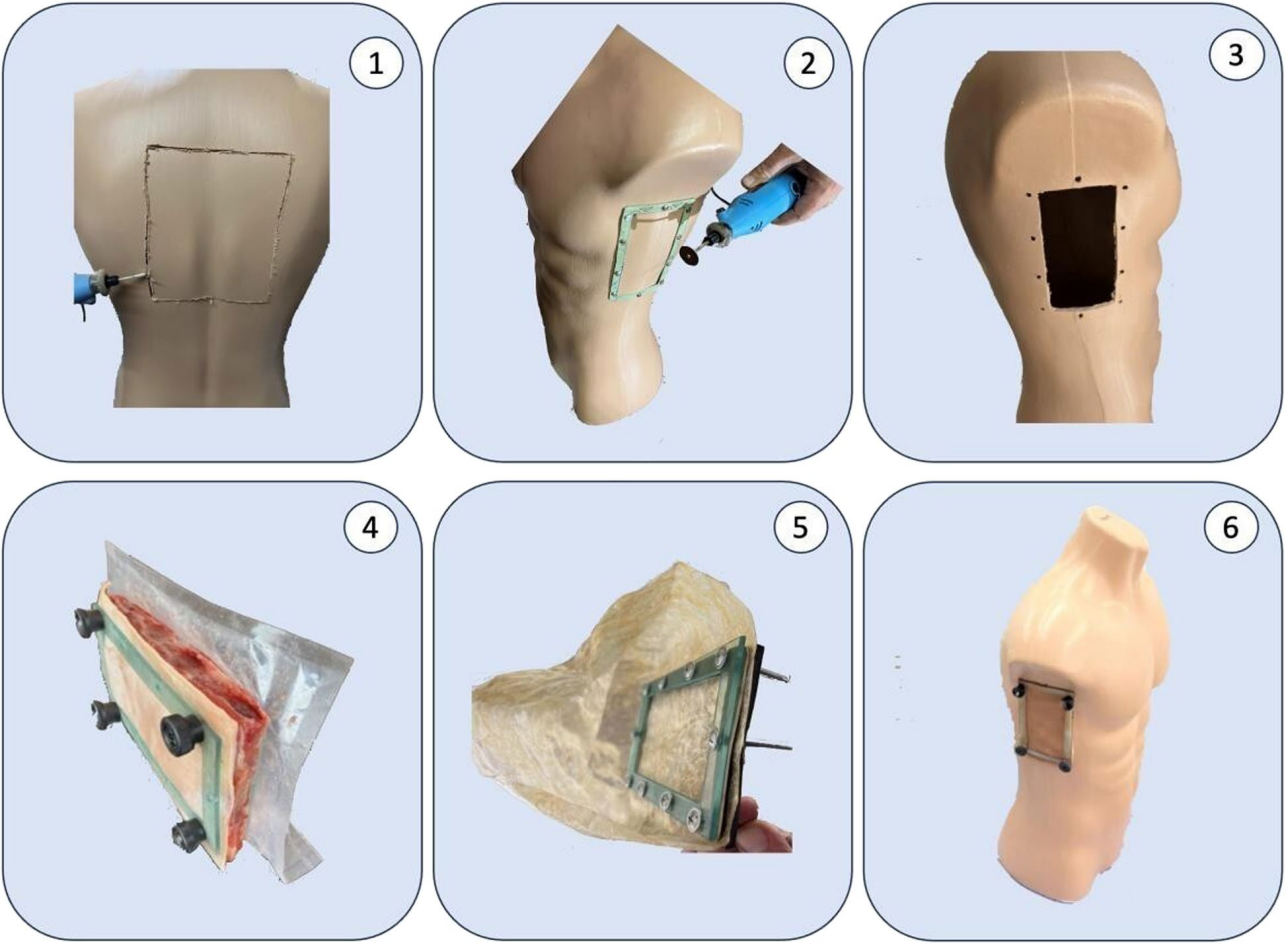
Assembly of the simulator, Bauru, 2023

The assembly was fixed to the wall with two acrylic plates (inner and outer) fixed with screws and nuts with threaded knobs. The EVA sealing gasket was cut to the same shape as the window to ensure the necessary seal during punching. The pressure of the screws on the acrylic, the EVA, the collagen, the wall of the mannequin, the rib, and the skin provided the necessary seal to prevent leaks during the punctures (Fig. 2).

**Fig. 3:**
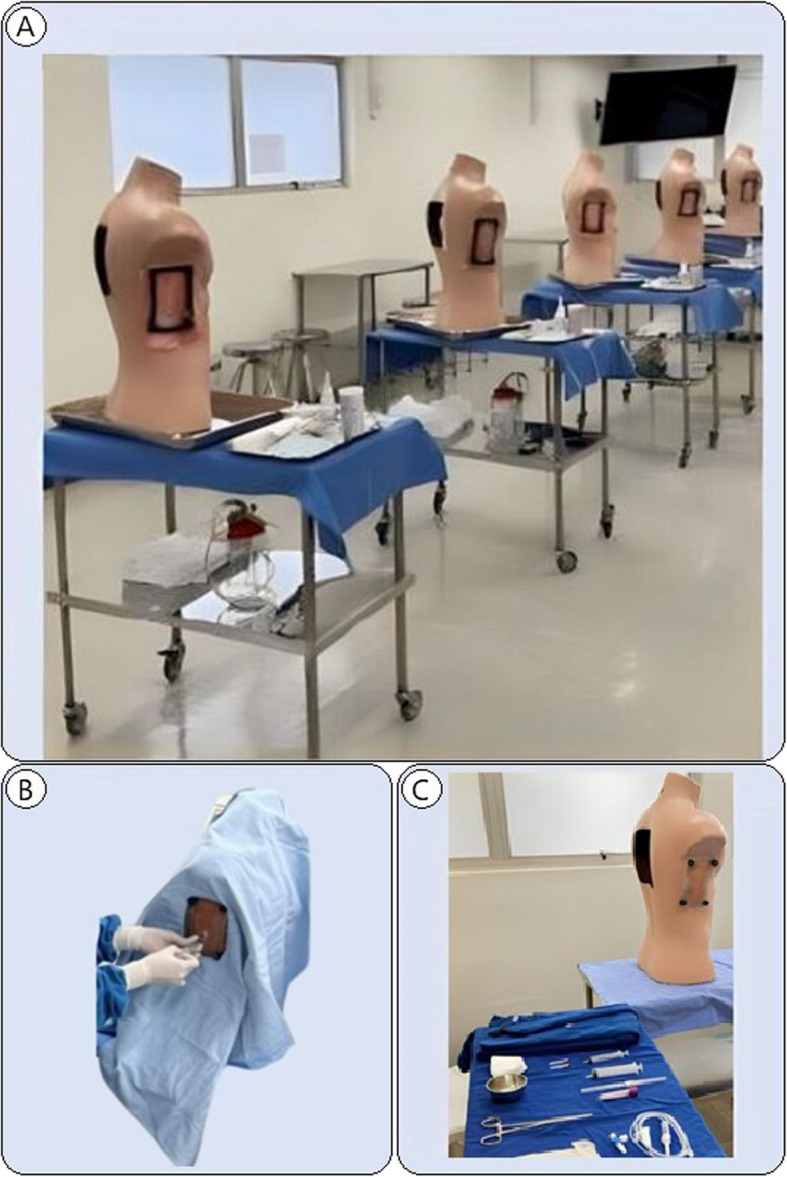
Assembled simulators, Bauru, 2023

The simulators developed were placed on a surgical table with the fields and materials needed to practice the thoracentesis procedural technique. The students had the opportunity to train their skills, take part in a clinical simulation scenario, and evaluate the technologies developed (Fig. 3).

### Face and content validation by experts and the pilot study

The validation of the thoracentesis scenario and simulator was carried out with the collaboration of three judges who had the following sociodemographic characteristics: specialization in general surgery (66.6%) and thoracic surgery (66.6%), with an average time working in the area of 17.6 years (minimum of 10 years and maximum of 30 years), with a doctorate (66.6%), experience in clinical simulation (100.0%) and scientific production in the area of surgery and simulation (100.0%).

Regarding the simulator, there was 100% agreement between the experts in the first evaluation process. Concerning the proposed scenario, adjustments were made and, after the second round of evaluation, there was 100% agreement on all the items in the scenario. Table 2 shows the items modified in the scenario.

**Table 2:** Items assessed and suitable for the proposed simulated clinical scenario, Bauru, 2023

Items assessed	Round 1	Round 2	Modified items
	CVI	CVI	
Primary objective	100.0%	100.0%	-
Secondary objectives	80.0%	100.0%	Adjusting verbs, withdrawal of inspection, palpation and percussion.
Target audience	100.0%	100.0%	-
Theoretical foundation	100.0%	100.0%	-
Material resources	100.0%	100.0%	-
Actor and simulator characterization	100.0%	100.0%	-
Clinical case	60.0%	100.0%	Taken from the physical examination inspection, palpation and percussion
Complementary tests	70.0%	100.0%	Printed complementary tests introduced (X-ray and full blood count), given to the student when requested. Leukocytosis, replaced by leukocytosis with left shift.
Student case description	100.0%	100.0%	-
Evaluation	90.0%	100.0%	*Objective Structured Assessment of Technical Skill *(OSAT) included

The pilot study consisted of skills training and scenario development. It took place in the clinical simulation laboratory and was complemented by debriefing, feedback, and student evaluation. Ten 4 th year undergraduate medical students took part in this stage of the pilot study. Among the students, five (50.0%) were female, and five (50.0%) were male; six (60.0%) reported that they carried out training in the skills and simulation laboratory voluntarily and as a complement to their undergraduate activities.

The students considered the clinical case to be well formulated, and the simulator was considered realistic by eight of them (80.0%). However, they pointed out that there was too much information, indicating that this could jeopardize the execution of the tasks set out in the scenario. They therefore suggested improvements in clarifying the clinical case, the inclusion of printed exams, and greater objectivity in the information and instructions on the tasks to be carried out. Table 3 shows the clinical simulation scenario in its final version, after the inclusions and exclusions suggested by the judges and students.

**Table 3:** Scenario of patients with pleural effusion, according to the criteria proposed by Fabri et al (2019)

✓ Target audience
3rd-year medical students
✓ Primary objective The student is able to: Indicate and perform thoracic drainage in a patient with purulent pleural effusion ✓ Secondary objectives. The student is able to: Identify the diagnosis and stratification of the type of parapneumonic effusion Perform the thoracentesis procedure: choosing the necessary materials, positioning the patient, defining the puncture site, anesthesia, procedure sequence, collecting material for analysis Perform respiratory physical examination techniques for patients with pleural effusion: auscultation
✓ Resources needed
Simulated patient, a pair of shorts or jeans, a short-sleeved shirt, a pair of trainers, artistic make-up, an identification bracelet, a stethoscope, a complete venturi mask, an oxygen mask with and without reservoir, an oxygen catheter, a laryngeal mask, mask valve bag, a low-fidelity thoracentesis simulator, a low-fidelity chest drainage simulator, procedure gloves, sterile gloves, sterile drapes, sterile apron, thoracentesis kit (antiseptic solution, gauze, glass with 1% lidocaine, 20mL syringe, aspiration needle, anesthesia needle, Jelco nº14, three-way stopcock, equipment, 3 sterile tubes for sample collection, collection bag), thoracic drainage kit, reclining stretcher, multiparametric monitor
✓ Characterization of the actor
The actor/patient is characterized by shorts or jeans, a short-sleeved shirt and trainers. In the scene, he/she is sitting up in bed, with an altered respiratory rate and paleness. Use artistic make-up to simulate the clinical condition.
Information for students You're a third-year student doing your internship in Internal Medicine at a tertiary hospital. You have been called to the ward to see a 42-year-old man who was admitted to the Emergency Department complaining of shortness of breath and chest pain for two days; he had already sought care at the Emergency Care Unit about two weeks previously for a fever and cough with expectoration. On admission, he was examined, additional tests were ordered and hospitalization was requested. Re-evaluate the patient, carry out a targeted anamnesis and physical examination interpret the tests requested, define the main diagnostic hypothesis, and carry out the necessary behavior to manage the individual's clinical condition. The patient's current vital signs: Blood Pressure = 130x90 millimeters of mercury; Oxygen saturation = 94%; Respiratory rate = 24 breaths per minute; Heart rate = 110 beats per minute; Temperature = 38.3°C
Chest X-ray Source: researcher's personal collection
**Guidance for simulated patients** You are a 42-year-old patient called Gustavo and you are in hospital for treatment. You've been married for 20 years, are the father of 2 children, from Lins, and come from Bauru. You sought medical attention about a fortnight ago, complaining of a cough, "wheezing" and fever (not measured); you were given medication (antibiotics), but stopped taking it on the 3rd day of treatment, after feeling better. At the reassessment, he will report that he feels tired, feverish and has chest pain, as well as difficulty breathing, which is accentuated when walking, and has worsened since he arrived at the hospital, making it difficult to speak. When asked about some aspects of the history of your current illness, you should verbalize the following information: ✓ About his symptoms = they started a fortnight ago, with a cough with phlegm and fever, which improved at the start of treatment, and two days ago he began to feel short of breath, which worsened when he made any physical effort ✓ About her diagnosis the first time she sought help = "They said I had a lung infection" ✓ About the treatment = he took it well for the first three days but stopped when his symptoms improved ✓ About personal history = has been smoking since the age of 20, from 1 to 2 packets a day, and drinks socially at weekends; does not use continuous medication or illicit drugs ✓ About his condition at the moment = he feels weak/tired, feverish, and short of breath, which has worsened since he arrived at the hospital, and at the moment even makes it difficult to speak ✓ About the pain = it's a pain of moderate intensity (6/7 on a scale of 1 to 10), which presents itself during breathing, especially on inspiration ✓ Other information = not in the script/not relevant ✓ During questioning, you should speak in a paused, lowered voice, followed by attempts at deeper breathing, which may or may not be followed by an expression of pain, and phrases such as "It's hard to even talk, Doctor".
✓ Evolution of the scenario:
If thoracentesis is indicated, offer the simulator to the student
✓ Evaluation
Procedure *checklist* and OSAT [60]
References
[12, 26–32]

## Discussion

The teaching of medical skills, especially procedural techniques such as chest drainage, faces significant challenges in a context where the need to balance practical training with patient and health professional safety is a pressing issue. To this purpose, the adoption of teaching methods that promote technical competence without exposing patients to unnecessary risks becomes crucial [36].

Clinical simulation is a teaching method that takes patient safety into account [37]. Well-structured simulated practice activities have an impact on student motivation [38] and enable not only the acquisition of diagnostic and therapeutic skills but also the development of competencies such as decision-making, teamwork, and effective communication [9]. The resources for clinical simulation sessions include simulators.

Simulators are technologies designed to reproduce and/or represent a device, body part, or service [37]. Simulators and simulated patients contribute to the emotional preparation of students, as they allow them to practice their skills in planned activities, allowing feelings such as anxiety and stress to be worked through, as well as enabling better learning to be achieved [39].

In general, low-cost simulators are usually developed using alternative materials that are easy to acquire and cost less than the reference models available on the market [36, 40–42].

The literature does not determine that more realistic simulators contribute more to learning than a low-fidelity simulator [43–45] what determines the resource to be used is always the learning objective of the activity [45]. Therefore, depending on the learning objectives set, the use of low-cost simulators can guarantee the effectiveness of simulation in the context of the teaching and learning process [42, 46–48].

Low-cost simulators, although less sophisticated in terms of technology when compared to robotic simulators, are capable of providing significant learning experiences when incorporated into simulated scenario contexts, allowing procedures to be repeated and errors to be corrected in a controlled environment and without risk to real patients, at a low cost to educational institutions [47, 49].

From a conceptual point of view, low-cost simulators have essential attributes, namely: cost, accessibility, technologies, manufacture and reproducibility, realism, versatility, and usability. In this study, the simulator was built based on three of these: realism, reproducibility, and cost.

As such, the simulator built has three fundamental characteristics: low-tech, handmade, and low-cost. It is classified as low-tech because it does not have technological resources such as electrical and electronic devices, software, or advanced technology. It can also be assembled intuitively. It was produced by hand, i.e. in a non-industrial environment, with hand tools and accessible materials, and in low demand. Finally, the low cost, given that industrial simulators for the same purpose cost much more than the one produced by the research team.

The simulator that has been built enables training in thoracentesis procedural skills. It was well evaluated by experts and students in the pilot project. It can also be reproduced for use in other procedures such as cystostomy, paracentesis, and jugular puncture, among many others, and can also be used for training and assessing professionals.

In addition to the industrial materials used to build the simulator in the report, the authors chose to include animal tissue (pig skin and ribs). However, these materials can be replaced with synthetic skin, easily made from silicone rubber, as these are more sustainable and bioethically adequate alternatives.

Other studies have also reported on the use of fruit, meat, and animal tissue as an alternative to increasing the realism of simulated training [40, 50, 51]. A study carried out in 2019 in Brazil presented the construction of two simulators: one for venous access and one for renal biopsy. The simulators were built with chicken breast, Penrose drain, plastic straw, and pig kidney. The authors report that the simulators allowed immediate identification of anatomical structures of interest and enabled the development of skills needed to perform invasive procedures [50].

Another Brazilian study developed a training model using tomatoes to acquire ophthalmological microsurgical skills. The models developed proved to be viable for training microsurgical dissection [51].

A study, also carried out in Brazil, developed a low-cost simulator for teaching the repair of obstetric anal sphincter injuries. In addition to other materials, beef was used. The simulator allowed participants to improve their knowledge of anatomy and physiology, and to develop surgical competencies and skills [40].

It’s important to note that the literature points to a tendency to classify simulators that use biological materials as handmade simulators. However, the authors of this article believe that “handmade” refers to the production method. In this way, low-cost simulators can be produced in an artisanal, industrial, or mixed way.

Artisanal simulators are developed and produced in a non-industrial environment, using manual and/or mechanical equipment and tools, with alternative and accessible materials, produced to specific demands, on a low scale, or an experimental basis.

It is also worth pointing out that, in terms of cost, considering the characteristics of perishable foods and the need for continuous replacement, simulators that use prime meats can have a high cost. Furthermore, low or high cost can have different connotations. A “low cost” in developed countries can be considered a “high cost” in developing or underdeveloped countries [42].

In this study, the simulator had a final cost of 18 US dollars, characterizing it as low-cost. It must be mentioned that the cost of materials may vary between different economic contexts — especially when comparing developed and developing countries, which may alter affordability.

A similar simulator, developed by Oliveira et al*.* in the city of Fortaleza, Brazil, cost US$21.00. In addition, the results point to its easy reproducibility and usefulness for teaching chest drainage [52]. In contrast, our study outperforms in describing transparently the simulated scenario and the steps in the material assembly process, in better differentiating the skin and muscle layers by using pork skin and EVA instead of only using EVA, and in enabling a more practical fluid refill.

A study carried out in southern Brazil by Bettega et al*.* with 49 medical students compared student safety in the closed chest drainage procedure in a low-cost model (synthetic, 3D printed) with an animal model (pork ribs). Although a higher score was observed in the synthetic model group for learning the chest drainage technique when compared to the animal model group, there was no statistical significance between the groups. There was a preference for the 3D model [53]. Despite that, not enough information was given about how the parts to build the simulator were assembled, hindering its reproducibility.

Some other low-cost simulators were validated for teaching chest drainage and thoracentesis. Yon et al*.* designed a model that heavily relies on adhesive materials, making it difficult to modify components [17]. Additionally, the anatomical accuracy could be hindered due to the large space allocated for the liquid reservoir. Talley et al. developed a simulator composed almost entirely of biological materials, rendering it highly perishable [18]. Young et al. exhibits assembly steps that require repeated replacement of adhesive materials, which may degrade the simulator over time [19]. Kouyoumjian et al. proposed a simulator in which its basic structure consists of a hamper and a pillow, putting visual and tactile fidelity into question [20]. Crawford et al. do not account for fluid removal training and replicate only a partial view of the rib cage [22].

Judges with expertise in surgery evaluated the clinical simulation scenario. In the first round of evaluation, only four criteria did not obtain a CVI of 100.0%. Therefore, the researchers decided to make the adjustments shown in Table 1 and send the scenario for another round of evaluation. After two rounds of evaluation, all the items reached 100.0% agreement. In addition, the scenario was also evaluated by students in a pilot study, adding clarification and complementary resources.

A clinical simulation scenario is an extremely important tool for planning, conducting, and evaluating simulated clinical experiences. A well-structured scenario with clear learning objectives can result in meaningful experience and learning [24, 54–56]. With this purpose, as presented in the results and suggested by the experts, they should be constructed with clear language and incorporate significant data into their execution.

The use of technologies such as the low-cost simulator and the scenario evaluated in this study can be considered a relevant cost-effective strategy for training healthcare professionals and, as highlighted in this research, future doctors.

Some limitations must be considered in this study. Ideally, thoracentesis is to be performed targeting the mid-axillary line for patients in the supine position or the posterior mid-scapular line for patients in the sitting position [57], with the assistance of an ultrasound device [58]. Although the sitting position was assumed in our scenario, the rubs were in placed in the mid-axillary line because it was later reutilized for thoracic drainage training. All participants were advised previously about this adaptation to the conventional technique, and about the recommended use of ultrasound. We considered that this would not compromise the training of thoracentesis. Also, a relatively small sample size (only 10 medical students in the pilot study and three experts) may limit the generalizability of the findings, especially those related to the use of Delphi method, which recommends a minimum of 12 participants [59]. These limitations must be addressed in future studies to validate the simulator and the scenario proposed in this study.

## Conclusions

A low-tech, handmade, low-cost simulator was built and evaluated for training in the procedural technique of thoracentesis and a clinical simulation scenario for managing patients with pleural effusion.

Although this study has some limitations, such as the small number of judges and the lack of evaluation of the simulator’s usability, which will be carried out in a later study, it was noted that the simulator had a much lower final cost than other models available in the industry. The desired realism was achieved in that it allowed thoracentesis and pleural drainage techniques to be carried out with sensory and visual characteristics that were very close to reality. Furthermore, the simulator parts can be built and replaced after multiple uses. Therefore, it can be reproduced easily with few resources.

In this study, the simulator built and the scenario presented were evaluated and considered suitable by experts in the field. They were also considered suitable by users (students). We therefore encourage the incorporation of these teaching resources, as well as future studies that can further expand the understanding of the impacts of their use and the learning of clinical skills.

## Data Availability

Data is available upon request from the first author.

## Abbreviations

EVA: Ethylene–vinyl acetate
CVI: Content Validity Index
